# The Unfolded Protein Response and Autophagy as Drug Targets in Neuropsychiatric Disorders

**DOI:** 10.3389/fncel.2020.554548

**Published:** 2020-09-29

**Authors:** Vignesh Srinivasan, Laura Korhonen, Dan Lindholm

**Affiliations:** ^1^Medicum, Department of Biochemistry and Developmental Biology, Faculty of Medicine, University of Helsinki, Helsinki, Finland; ^2^Minerva Foundation Institute for Medical Research, Biomedicum Helsinki 2U, Helsinki, Finland; ^3^Department of Biochemical and Clinical Sciences (BKV), Linköping University, Linköping, Sweden; ^4^Department of Child and Adolescent Psychiatry, Region Östergötland, Linköping, Sweden

**Keywords:** UPR, autophagy, synapse, neuropsychiatric disease, drug

## Abstract

Neurons are polarized in structure with a cytoplasmic compartment extending into dendrites and a long axon that terminates at the synapse. The high level of compartmentalization imposes specific challenges for protein quality control in neurons making them vulnerable to disturbances that may lead to neurological dysfunctions including neuropsychiatric diseases. Synapse and dendrites undergo structural modulations regulated by neuronal activity involve key proteins requiring strict control of their turnover rates and degradation pathways. Recent advances in the study of the unfolded protein response (UPR) and autophagy processes have brought novel insights into the specific roles of these processes in neuronal physiology and synaptic signaling. In this review, we highlight recent data and concepts about UPR and autophagy in neuropsychiatric disorders and synaptic plasticity including a brief outline of possible therapeutic approaches to influence UPR and autophagy signaling in these diseases.

## Introduction

Neuropsychiatric disorders confine illnesses and symptoms that are associated with brain abnormalities (Yudofsky and Hales, 1989). Patients with these disorders often have disturbances in the regulation of their mood, emotions, social behavior, and cognitive abilities such as memory, thought process, inhibition, and attention. These symptoms reflect structural and functional abnormalities that arise during the development or due to neurological diseases such as neurodegenerative disorders, stroke, and traumatic brain injury.

Major neurodevelopmental disorders encompass autism spectrum disorders (ASD), Attention deficit hyperactivity disorder (ADHD), Tics/Tourette’s syndrome (TS), developmental coordination disorder, communication disorders, specific learning disorders, and intellectual disability (IF; Thapar et al., 2017). Furthermore, schizophrenia, rare genetic syndromes, and congenital neural anomalies can be included in a broader definition of neurodevelopmental disabilities (Rapoport et al., 2005; Weinberger, 2017).

Neurodevelopmental disorders are most often heritable and multifactorial, implying that both genes and non-heritable factors contribute to the disorders (Doherty et al., 2018). The overlap between different disorders and their constituent symptoms is high (Gillberg, 2010). Also, neurodevelopmental disorders are often comorbid with other psychiatric disorders such as mood disorders and anxiety (Merikangas et al., 2015; King, 2016). Psychiatric symptoms are also present in many neurological disorders. For example, depression and anxiety are common in different neurodegenerative disorders such as Parkinson’s disorder (PD), Alzheimer’s disease (AD), and Huntington’s disease (PD), as well as after stroke and traumatic brain injury (Ayerbe et al., 2013; Arciniegas and Wortzel, 2014; van Duijn et al., 2014; Zhao et al., 2016; Seppi et al., 2019; Sellers et al., 2020).

At the cellular level, neurological disorders have been associated with dysfunctional protein and intracellular organelle homeostasis resulting in defective neuronal signaling and synaptic events. Protein quality control constitutes proper protein folding and modifications at the endoplasmic reticulum (ER) after synthesis, as well as fine-tuned protein turnover and degradation *via* the ubiquitin-proteasome system, and autophagy machinery. Neurons exhibit a high degree of complexity and regulate these processes locally in different sub-compartments. In line with this, defects in the functioning of the ubiquitin-proteasome system, ER signaling, and autophagy have been linked to the pathology of neuropsychiatric disorders (Martínez et al., 2018; Tomoda et al., 2019; Luza et al., 2020).

Currently, no curative medication is available for any of the neuropsychiatric disorders. Thus, current treatment guidelines recommend psychosocial interventions and if needed symptom alleviating medication. Novel insights into mechanisms are required to spur drug development. In the following sections, we will briefly review the current knowledge about the unfolded protein response (UPR) and autophagy in models of neuropsychiatric diseases and the possibilities for drug interventions and benefits for future treatments.

## The Unfolded Protein Response

**Figure 1:**
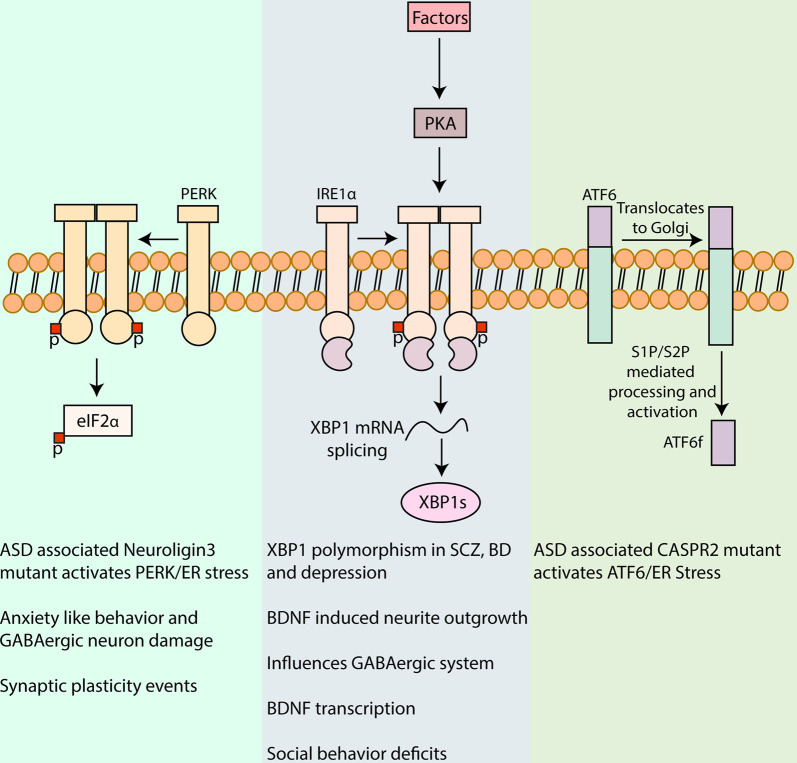
Involvement of unfolded protein response (UPR) in neuropsychiatric disorders. UPR is a major pathway in protein quality control in cells including neurons and is characterized by signaling cascades in the ER, mediated by the protein sensors, PERK, IRE1α, and activating transcription factor 6 (ATF6). Activation of PERK by autophosphorylation leads to phosphorylation of eukaryotic initiation factor 2 α (eIF2α), reducing the synthesis of critical synaptic proteins in neurons that affects memory processes and synaptic plasticity associated with intellectual disability, cognition, and addiction (Trinh et al., 2014; Placzek et al., 2016). PERK/eIF2α has also been linked to the pathophysiology of autism spectrum disorder (ASD) and mutant Neuroligin3 induced UPR and to anxiety-like behavior and GABAergic neuron damage in the amygdala (Ulbrich et al., 2016; Trobiani et al., 2018). IRE1α upon activation dimerizes and auto-phosphorylates which activates its kinase and RNase domain. The RNase domain further performs the unconventional splicing of XBP1 mRNA, resulting in spliced XBP1 mRNA that translates into a potent transcription factor, XBP1s (Uemura et al., 2009). XBP1 polymorphism in its promoter region has been associated with, Schizophrenia (SCZ), bipolar disorder (BD), and depression (Kakiuchi et al., 2003; Watanabe et al., 2006; Grunebaum et al., 2009). XBP1s also upregulates brain-derived neurotrophic factor (BDNF) that is a major factor involved in synaptic plasticity in health and disease. Activation of Protein kinase A (PKA) *via* cyclic AMP by different agents in the brain can in turn influence IRE1α (Saito et al., 2018). Inhibition of IRE1α ameliorated the social behavior deficits, a commonly observed trait in some neuropsychiatric disorders (Crider et al., 2018). Upon UPR induction, ATF6 is translocated to the Golgi apparatus for further processing by Site-1 and Site-2 proteases (S1P and S2P, respectively), leading to the release of amino-terminal fragment ATF6f with transcription factor functions. The expression of a mutant CASPR2 associated with ASD was shown to increase ATF6 (Falivelli et al., 2012; Canali et al., 2018). The precise role of ATF6 signaling in neuropsychiatric disorders warrants further investigations.

UPR is crucial for protein quality control in all cells and has a protective function in coping with cell stress and the accumulation of misfolded or mutant proteins in the endoplasmic reticulum (ER). In neurons, UPR is essential for the maintenance of neuronal adaptive capacity to the varying growth and stress stimuli experienced over their lifetime. UPR is involved in the pathophysiology resulting from misfolded proteins like Tau, α-Synuclein, and Huntingtin associated with neurodegenerative diseases (Hetz and Saxena, 2017; Lindholm et al., 2017). A growing body of evidence has also indicated the importance of UPR in neuronal signaling and in neuropsychiatric disorders characterized by neurodevelopmental and synaptic deficits (Martínez et al., 2018). Initiation of UPR results in a set of responses aimed towards reducing the proteostasis burden on ER machinery by halting protein translation and increasing the protein folding capacity *via* transcriptional induction of chaperones and components of ER machinery. In some cases, the proteostasis burden on the ER exceeds the limit manageable and leads to apoptotic signaling as observed in neurodegenerative and other diseases (Lindholm et al., 2017). Three main transmembrane receptors are present in the ER, inositol-requiring protein 1α (IRE1α), protein kinase RNA-like endoplasmic reticulum kinase (PERK), and activating transcription factor 6 (ATF6) that become activated during the UPR (see Figure 1). The chaperone, Binding-immunoglobulin protein/78 kDa glucose-regulated protein (BiP/Grp78) is normally associated with the luminal parts of these proteins, keeping them in their inactive state. Upon accumulation of mutant or misfolded proteins in the ER lumen, BiP is released and the different receptors are activated on a certain time scale (Lindholm et al., 2017; Martínez et al., 2018). This culminates in the transcriptional reprogramming of the cell to combat the ensuing ER stress (Han and Kaufman, 2017). Also, the UPR plays a role in neuronal signaling and synaptic neurotransmission. Most of the therapeutic strategies developed against ER stress have focused on modulating the UPR signaling in neurodegenerative disorders like HD, Parkinson’s disease (PD), and Amyotrophic Lateral Sclerosis (ALS; Hetz and Saxena, 2017; Lindholm et al., 2017). Recent studies have advanced our knowledge of the importance of UPR in the pathophysiology associated with neuropsychiatric disorders. The following provides a brief account on the functioning of UPR signaling cascades and their involvement in neuropsychiatric disorders.

## UPR in Neuropsychiatric Disorders

Synaptic modulation requires a constant turnover of specific proteins and vesicles involved in the regulation of neurotransmission. The UPR *via* effects on protein synthesis is indirectly involved in the regulation of synaptic architecture and neuronal signaling. Mutations in the synaptic proteins, neuroligin3, and contactin-associated protein-like 2 (CASPR2), cause retention of these misfolded proteins in the ER that is associated with ASD (Ulbrich et al., 2016; Canali et al., 2018). Induction of the UPR or its signaling cascades have been linked to pathologies associated with neuropsychiatric and cognitive deficits like memory consolidation defects; ASD; schizophrenia; post-traumatic stress disorders; stress-induced mental disorders; bipolar disorder; and impaired social behavior (Grunebaum et al., 2009; Falivelli et al., 2012; Di Prisco et al., 2014; Ulbrich et al., 2016; Wen et al., 2016; Crider et al., 2017; Dong et al., 2018; Shen et al., 2019). The precise mechanisms and molecules contributing to the pathophysiology of these neuropsychiatric disorders are incompletely understood.

## PERK Signaling

Upon UPR induction, PERK undergoes oligomerization and phosphorylation that in turn activates the eukaryotic initiation factor 2α (eIF2α). Phosphorylated eIF2α inhibits the translation of proteins to reduce the protein load on the ER. On the other hand, the translation of specific mRNAs like ATF4 with an upstream open reading frame in their 5′ UTR is increased (Cnop et al., 2017). ATF4 encodes a transcription factor leading to an upregulation of genes like C/EBP Homologous Protein (CHOP), Growth arrest and DNA damage-inducible protein (GADD34), and ER protein folding chaperones, in addition to those associated with metabolic regulation (Han and Kaufman, 2017). GADD34 is a protein phosphatase that dephosphorylates eIF2α, establishing a feedback loop in UPR signaling, and restoring protein synthesis (Hetz and Papa, 2018). Also, PERK can phosphorylate nuclear factor erythroid 2-related factor 2 (Nrf2) to regulate oxidative stress responses (Cullinan and Diehl, 2004).

PERK/eIF2α signaling plays an important role in regulating neuronal protein synthesis and synaptic plasticity. Thus, inhibiting PERK locally in the hippocampus, using the compound GSK2606414 improved memory in young mice, while ameliorating memory defects in aged mice (Sharma et al., 2018). In neuronal PC12 cells, expressing the autism-linked mutant R451C neuroligin3 PERK and other UPR signals were activated (Ulbrich et al., 2016). In a mouse knock-in model for this mutant neuroligin3, UPR was activated specifically in the cerebellum with an increased excitatory current in Purkinje cells, that was restored to normal levels upon PERK inhibition (Trobiani et al., 2018). More studies are required to understand how the mutant neuroligin3 induced UPR activation is related to ASD associated synaptic pathologies. PERK has also been linked to stress-induced anxiety, and reducing PERK was found to mitigate ER stress-induced GABAergic neuron damage in rat basolateral amygdala (Wang S. et al., 2019).

Studies employing gene deleted, knock-out (KO) mice for PERK have been particularly rewarding to reveal its role in synaptic plasticity and functions. Hippocampus late long-term potentiation (L-LTP), inhibited by rapamycin acting on the mammalian target of rapamycin complex 1 (mTORC1), was reversed in the PERK KO mice. Mechanistically, the PERK KO mice displayed no alterations in mTORC1 signaling but showed reduced phosphorylation of eukaryotic elongation factor 2 (eEF2) with increased protein translation (Zimmermann et al., 2018). These results point to the role of PERK in mTORC1-independent L-LTP *via* its effects on eEF2-mediated translation.

In another study, the deletion of PERK in the mouse forebrain resulted in decreased p-eIF2α and ATF4 levels accompanied by impairments related to information processing and behavior flexibility in these mice. The levels of PERK and ATF4 were also reduced in the frontal cortex in postmortem samples from schizophrenic patients (Trinh et al., 2012). Together these results indicate a role for PERK in cognitive functions and behavioral responses. In the forebrain specific PERK KO mice, the metabotropic glutamate receptor-dependent long-term depression (LTD) was specifically enhanced, suggesting involvement of PERK in this form of synaptic plasticity (Trinh et al., 2014). The role of PERK-eIF2α signaling in synaptic functions might be context-dependent and involve eIF2α, as well as other factors. In midbrain dopamine neurons, eIF2α-mediated translational control can regulate cocaine-induced LTP in a model of drug addiction (Placzek et al., 2016).

Mice lacking the Ca_v_1.2 subunit of L-type Ca^2+^ channel (CACNAC1C) in the forebrain exhibited social behavior deficits and a higher excitatory/inhibitory (E/I) ratio, a characteristic of patients with schizophrenia and ASD. Detailed analysis showed a decrease in protein synthesis, mTORC1-dependent translation factors, and an associated increase in p-eIF2α levels. Treatment of the mice with the integrated stress response inhibitor (ISRIB) inhibiting p-eIF2α, normalized protein synthesis, the E/I ratio, and reversed the social deficits (Kabir et al., 2017). Together these results indicate that PERK/eIF2α-mediated protein translational control regulates synaptic plasticity and is associated with pathologies accompanying neuropsychiatric disorders.

## IRE1α Signaling

The activation of IRE1α can occur *via* two models, either by the dissociation of BiP upon the accumulation of misfolded proteins in the ER lumen (Amin-Wetzel et al., 2019) or by binding of misfolded proteins to the luminal part of IRE1α (Martínez et al., 2018). Upon activation, monomeric IRE1α dimerizes resulting in autophosphorylation of its kinase domain. This phosphorylation changes the protein conformation, activating the endoribonuclease domain (RNase domain) of IRE1α located on the cytoplasmic side of the ER membrane. This results in the unconventional splicing with the removal of 26 nucleotides in the unspliced X-box binding protein 1 mRNA (XBP1u) producing a novel mRNA species that encodes a transcription factor, spliced X-box binding protein 1 (XBP1s; Uemura et al., 2009). XBP1s is a potent transcription factor, which upon translocation to the nucleus binds to promoter regions containing ER stress response elements (ERSE). XBP1s drives the transcription of its mRNA, forming a positive feedback loop. In eukaryotes, XBP1u mRNA can still be translated into a protein with less stability that is rapidly degraded by the ubiquitin-proteasome system (Navon et al., 2010). Our recent work showed that proteasome inhibition, stabilizing the protein, resulted in the formation of XBP1u aggresome like induced structures in neuronal cells (Srinivasan et al., 2020). The role of these XBP1u structures in neuronal IRE1α signaling remains to be explored.

The importance of IRE1α/XBP1 in neuropsychiatric disorders was revealed by the identification of a single nucleotide polymorphism (SNP) in the promoter region of the XBP1 gene (116C→G substitution) in patients afflicted by bipolar disorder. Molecular details showed that the polymorphism reduced the tunicamycin-induced XBP1 expression in lymphoblastoid cells derived from bipolar patients, compared with controls (Kakiuchi et al., 2003). The association of XBP1 polymorphism to schizophrenia was also observed in the Japanese population, but the results were not confirmed by another study (Watanabe et al., 2006). Also, XBP1 polymorphism was linked to the clinical course of major depressive episodes and elevated morning plasma cortisol in a subgroup of patients (Grunebaum et al., 2009). Together these studies show a correlation between IRE1α/XBP1 signaling and certain mood disorders that warrant further investigations.

Mechanistic studies have shown that IRE1α/XBP1 signaling is linked to the action of the brain-derived neurotrophic factor (BDNF) that regulates synaptic plasticity and neuronal survival. BDNF increased XBP1 splicing in primary neuronal cultures, while the loss of XBP1 disrupted the BDNF induced neurite outgrowth (Hayashi et al., 2007). Also, BDNF mediated increases in GABAergic markers, including somatostatin, neuropeptide Y, and calbindin were reduced in XBP1 deficient neurons, suggesting a role of XBP1 in inhibitory neurotransmission (Hayashi et al., 2008). Mice lacking XBP1 showed memory deficits, and a reduced expression of memory associated genes and these were restored upon expression of XBP1s. Amongst the genes, BDNF was identified as a key transcriptional target of XBP1s (Martínez et al., 2016). Neuronal activity induced by glutamate increases IRE1α/XBP1 signaling in dendrites with an enhanced BDNF transcription, whilst the addition of recombinant BDNF treatment upregulated IRE1α/XBP1 (Saito et al., 2018). Protein Kinase A (PKA) was shown to be an important mediator in this process. This is of importance and indicates that several factors and agents acting *via* cyclic adenosine monophosphate (cAMP) and PKA in the brain might have a similar effect on IRE1α/XBP1 and requires further studies.

Social behavior is altered in several psychiatric and mood disorders and reflects changes in brain connectivity between the prefrontal cortex (PFC) and hippocampus. Tunicamycin treatment was found to activate IRE1α/XBP1 signaling in the PFC, whilst silencing of IRE1α ameliorated the social behavior deficits in mice. Furthermore, the administration of the Estrogen receptor β (ERβ) agonist, ERB-041 improved social behavior, attenuated the increase in functional connectivity between PFC and hippocampus, whilst reducing phosphorylation of IRE1α (Crider et al., 2018). Further studies are warranted to expand our understanding of the role of IRE1α/XBP1 in synaptic plasticity and its defects observed in neuropsychiatric disorders.

## ATF6 Signaling

Normally, ATF6 is associated with BiP keeping it in the ER compartment. Upon activation of UPR, ATF6 is translocated to the Golgi apparatus to undergo processing by the Site-1 and Site-2 proteases (S1P and S2P), releasing an N-terminal fragment (ATF6f; Lindholm et al., 2017; Martínez et al., 2018). ATF6f is then translocated to the nucleus to perform transcription factor functions regulating XBP1s and ER-associated degradation (ERAD) components. ATF6f can also form heterodimers with XBP1s to regulate an expanded repertoire of genes mediating crosstalk between the two branches of UPR signaling (Shoulders et al., 2013).

The potential role of ATF6 in neuropsychiatric disorders is only emerging (Wen et al., 2016; Crider et al., 2017). Mutations in CASPR2 associated with the occurrence of ASD causes axonal growth defects in mouse-derived primary cortical neurons (Canali et al., 2018). The mutant protein is retained in the ER after synthesis and initiates the UPR. ATF6 signaling was increased in cells transiently expressing mutant CASPR2 (Falivelli et al., 2012). Further studies are warranted to decipher the involvement of ATF6-mediated UPR signaling in synaptic defects and associated neuropsychiatric disorders.

## Targeting the UPR in Neuropsychiatric Disorders

As shown in Figure 1, UPR and its components have been implicated in the pathology associated with different neuropsychiatric disorders. There is still a large gap in translating the identified pathological mechanisms into therapies. Despite this, some drug candidates have been shown to modulate the UPR signaling, and thereby, raising the possibility to develop novel therapeutic strategies in neuropsychiatric disorders (Table 1).

**Table 1:** Drugs targeting the unfolded protein response (UPR).

Drug	Model	Outcomes	Reference
Valproate	Lymphoblastoid cells from patients with Bipolar disease.	Tunicamycin-induced activation of XBP1 reduced.	Kakiuchi et al. (2003)
Valproate	Mouse neuroblastoma N2A cells.	Increases in XBP1 mediated WFS1 expression, reduced WFS1-Grp94 interaction.	Kakiuchi et al. (2009)
Valproate	Primary neuronal cultures.	Increases in ER proteins, Grp78, Grp94 and Calreticulin. No cell death.	Shao et al. (2006)
Lithium
Fluvoxamine	HEK 293 and mouse neuroblastoma N2A cells.	ATF4-mediated increase in Sigma-1 receptor (S1R). Reduced ER stress and cell death.	Omi et al. (2014)
Haloperidol	Neuroblastoma-glioma NG-108 cells.	Binding to S1R and regulation of IP3R Ca^2+^ signaling.	Kubickova et al. (2018)
Olanzapine	SH-SY5Y human neuroblastoma cells Rat *in vivo* model.	Increases in PERK/eIF2α signaling *in vitro* and in rat hypothalamus.	He et al. (2019)
Trazodone hydrochloride	HEK 293 and mouse neuroblastoma N2A cells.	Inhibition of Tunicamycin-induced ATF4, independent of eIF2α.	Ii Timberlake and Dwivedi (2019)
Ketamine	Rat *in vivo* model.	mTOR-dependent increases in CHOP, IRE1α and PERK.	Abelaira et al. (2017)
Estrogen receptor β (ERβ) agonist, ERB- 041	Mouse *in vivo* model.	Amelioration of tunicamycin induced behavior deficits and IRE1α phosphorylation.	Crider et al. (2018)

Valproate and lithium are mood-stabilizing drugs employed for the treatment of bipolar disorders. As discussed above, lymphoblastoid cells derived from patients with bipolar diseases show reduced ER stress-induced XBP1 expression, which was restored by treatment with Valproate (Kakiuchi et al., 2003). Valproate also enhanced expression of the Wolfram syndrome protein (WFS1) in neuroblastoma N2A cells and reduced its interaction with the 94 kDa glucose-regulated protein (GRP94; Kakiuchi et al., 2009). Wolfram syndrome is associated with mental disorders as well as metabolic disturbances, and WFS1 is a component of the UPR regulating calcium homeostasis (Fonseca et al., 2005). GPR94 is an ER chaperone regulating the transport and processing of secreted proteins and in the regulation of ERAD that may associate with bipolar disease (Kakiuchi et al., 2009).

Lithium has been used as a mood stabilizer in bipolar disorders for a long time (Machado-Vieira, 2018). Lithium has multiple effects on cell signaling including, regulating intracellular calcium and cAMP levels, activation of PKA, and Protein Kinase C (PKC; Malhi et al., 2013; Limanaqi et al., 2019). In primary neuronal cultures, lithium increases the expression of the ER proteins, Grp78, Grp94, and Calreticulin (Shao et al., 2006) suggesting that bipolar disorders may involve changes in ER stress responses (Limanaqi et al., 2019). More credence to this view comes from findings that the mRNA encoding BiP was significantly increased, whilst total XBP1 (XBP1s + XBP1u) and XBP1u mRNAs were decreased in peripheral blood of patients with the bipolar disease compared with controls (Bengesser et al., 2018). This may support the view that the UPR is impaired in bipolar disorders indicating an important target for drug intervention.

The sigma-1 receptor (S1R) is localized to the mitochondria-associated ER membrane (MAM) compartment. S1R plays a vital role in neuronal physiology by regulating inositol 1, 4, 5-trisphosphate receptor (IP3R) involved in calcium homeostasis of ER and mitochondria. Owing to its wide range of protein interactions, S1R affects neuronal excitability, synaptic plasticity, and ER stress signaling (Ryskamp et al., 2019). The selective serotonin reuptake inhibitor, fluvoxamine was found to increase expression of S1R that mitigated cell demise induced by ER stress in neuroblastoma cells (Omi et al., 2014). Haloperidol, an antipsychotic drug blocking postsynaptic dopamine D2 receptors in the brain, binds with S1R and regulates IP3R signaling in neuroblastoma-glioma NG-108 cells (Kubickova et al., 2018). Taken together, S1R is a promising target to consider in neuropsychiatric disorders including schizophrenia, depression, methamphetamine, and cocaine addiction (Kourrich et al., 2012, 2013; Wang et al., 2016; Sambo et al., 2018; Soriani and Kourrich, 2019; Yang et al., 2019).

Regarding other drugs, it was shown that the antipsychotic compound, Olanzapine enhanced PERK/eIF2α signaling in the hypothalamus of rats that was linked to increased body weight. Treatment with the ER stress inhibitor, 4-phenylbutyrate (4-PBA) attenuated the effects brought about by Olanzapine (He et al., 2019). Trazodone hydrochloride is a serotonin reuptake inhibitor and an antidepressant medicine, reduced activating transcription factor 4 (ATF4) levels in cells treated with tunicamycin to induce ER stress (Ii Timberlake and Dwivedi, 2019). Also, ketamine, an NMDA receptor antagonist, increased the ER signaling proteins, IRE1α, PERK, and CHOP, in certain brain regions in male rats. Notably, these increases were related to the Mammalian target of rapamycin (mTOR) activation (see below) and abrogated by rapamycin indicating that the anti-depressant action of ketamine may involve UPR signaling (Abelaira et al., 2017). Further studies, using cell cultures and animal models, are useful to reveal drugs influencing UPR signaling in the brain and in neuropsychiatric disorders.

## Autophagy and Neuronal Activity

Autophagy is an evolutionarily conserved mechanism and is mediated *via* a complex network of over 30 proteins that are conserved across species. Post-mitotic neurons become highly dependent on autophagy for reducing their cytosolic load by clearing its components like misfolded proteins and dysfunctional organelles (Bar-Yosef et al., 2019). Recently, specific aspects of autophagy in different neuronal compartments have been thoroughly covered (Hill and Colon-Ramos, 2020), and we will mainly focus on the potential role of autophagy processes in neuropsychiatric disorders.

Autophagy is critical in maintaining neuronal functions by clearing cytoplasmic components and organelles including misfolded proteins, promoting turnover of gamma-aminobutyric acid (GABA) and α-amino-3-hydroxy-5-methyl-4-isoxazolepropionic acid (AMPA) receptors important for plasticity, recycling presynaptic proteins and synaptic vesicles, and regulating dendritic morphology and synaptic pruning in development and disease (Liang, 2019; Hill and Colon-Ramos, 2020). A failure in any of these processes may impair neuronal functions and cause neurodegenerative disorders (Bar-Yosef et al., 2019). Given this, several studies have focused on the therapeutic potentials of modulating autophagy in these disorders.

Synaptic autophagy is vital for the regulation of pre- and postsynaptic protein turnover and signal transmission. Proteins like Bassoon, EndophilinA (EndoA), and Synaptojanin-1 (Synj1) involved in synaptic transmission influence also autophagy in the presynaptic compartment. Bassoon present in the synaptic active zone can bind to the autophagy protein, autophagy-related 5 (ATG5) and reduce autophagy whereas, EndoA and Synj1, which both play a role in endocytosis, also induces synaptic autophagy and formation of autophagosomes (Okerlund et al., 2017; Liang, 2019; Tomoda et al., 2019; Hill and Colon-Ramos, 2020). Furthermore, several proteins in the postsynaptic compartment including postsynaptic density protein 95 (PSD-95), and SH3 and multiple ankyrin repeat domains 3 (SHANK-3) are targets for autophagy degradation (Nikoletopoulou et al., 2017). These findings support the view that there is an interplay between synaptic activity and autophagy in fine-tuning neurotransmission by modulating synaptic proteins (Nikoletopoulou and Tavernarakis, 2018).

In neurons, BDNF regulates synaptic plasticity by reducing autophagy flux in an mTOR dependent manner (Nikoletopoulou et al., 2017). BDNF binds preferentially to its high-affinity receptor Tropomyosin receptor kinase B (TrkB) that is retrogradely transported in autophagosomes together with BDNF to influence neuron-specific functions in the soma (Kononenko et al., 2017). BDNF like other neurotrophins can also interact with the p75 neurotrophin receptor (p75NTR). Nerve growth factor (NGF) was shown to regulate autophagy in cerebellar Purkinje cells in cultures *via* p75NTR (Florez-McClure et al., 2004). The precise role of p75NTR and the effects of different neurotrophins in regulating neuronal autophagy *in vivo* warrant further studies.

Recent studies performed in mice showed that autophagy is important for memory formation in the hippocampus. Autophagy activity was reduced during aging, whilst stimulation of autophagy by injection of the autophagy regulator Beclin-1 into the hippocampus, or using specific systemic factors reversed age-dependent memory deficits (Glatigny et al., 2019). The molecular mechanisms underlying these effects will require further studies. The accumulated results so far point towards a context-dependent role for autophagy in regulating synaptic plasticity and neuronal functions. Although a baseline level of autophagy is required for maintenance of synapse, excessive autophagy could be detrimental by depleting synaptic proteins. Together with other findings, these studies show that autophagy and its regulation play an important role in activity-dependent synaptic functions and in memory formation in the brain.

## Autophagy and Neuropsychiatric Disorders

**Figure 2:**
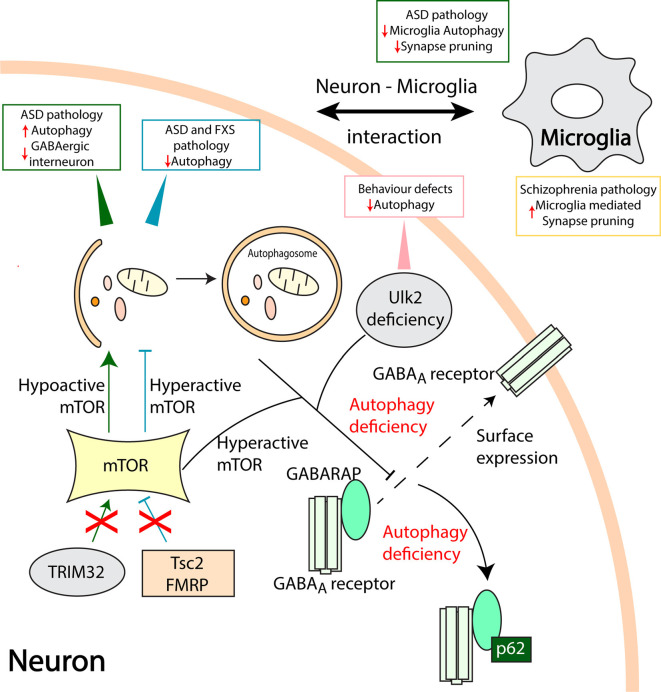
Aberrant autophagy processes in neuropsychiatric disorders. Mammalian target of rapamycin (mTOR) is a vital checkpoint in autophagy regulation. Hyperactive mTOR associated autophagy inhibition in Tuberous Sclerosis Complex 2 (Tsc2) and fragile X mental retardation protein (FMRP) deficient models result in ASD and Fragile X syndrome (FXS) pathologies, respectively (Tang et al., 2014; Yan et al., 2018). On the other hand, loss of TRIM32 leads to hypoactive mTOR and increased autophagy leading to ASD like pathology and loss of GABAergic interneurons (Zhu et al., 2019). Ulk2 deficiency causes autophagy inhibition and behavioral defects in rodents. Furthermore, hyperactive mTOR and Ulk2 deficiency mediated autophagy defects disrupt the gamma-aminobutyric acid (GABA) Type A Receptor-Associated Protein (GABARAP) mediated trafficking of GABA_A_ receptors to the plasma membrane (dashed line arrow; Sumitomo et al., 2018; Hui et al., 2019). An autophagy adapter protein, p62 sequesters GABARAP, and GABA_A_ receptors when autophagy is compromised. Also, communication between neurons and microglia is pivotal in maintaining healthy synapses. Microglia specific loss of autophagy results in ASD like behavioral deficits and reduced synaptic pruning whilst, microglia-mediated increase in synaptic pruning might be associated with the pathology of schizophrenia (SCZ; Sellgren et al., 2019). Direct involvement of microglia autophagy and synapse pruning in SCZ requires further studies.

Accumulating evidence also indicates that autophagy can play a role in neuropsychiatric disorders like major depressive disorders, ASD, and impaired cognition (Tang et al., 2014; Jia and Le, 2015; Bar-Yosef et al., 2019; Glatigny et al., 2019; Tomoda et al., 2019). Some of the mechanisms involved have been depicted in Figure 2.

mTOR is known to negatively regulate autophagy in non-neuronal as well as in neuronal cells in a complex and context-dependent manner. mTOR contributes to neurophysiological changes observed in models of ASD and fragile X syndrome (FXS). Tuberous Sclerosis Complex 2 (Tsc2) deficient mice exhibited enhanced mTOR activity resulting in reduced autophagy, impaired synaptic pruning, and an excessive dendritic spine formation (Tang et al., 2014). Autophagy flux is compromised in the hippocampus of the FXS mouse model and enhancing autophagy counteracted synaptic and cognitive impairment in these mice (Yan et al., 2018). The tripartite motif protein 32 (TRIM32) has emerged as a regulator of mTOR *via* proteasome degradation of G protein signaling protein 10 (RGS10). Gene deficient mice for TRIM32 showed increased autophagy and autism-like behavior with the involvement of GABAergic interneurons, whilst restoring autophagy to normal levels counteracts these effects (Zhu et al., 2019). Autophagy is further linked to GABAergic signaling *via* the GABA_A_ receptor-associated protein (GABARAP), a member of the autophagy-related protein 8 (ATG8) family of proteins required for autophagosome maturation (Weidberg et al., 2010; Schaaf et al., 2016). GABARAP affects the trafficking and surface localization of GABA_A_ receptor to the plasma membrane and these events were impaired in autophagy-deficient adult brain lacking the autophagy-related protein 7 (ATG7) gene (Hui et al., 2019).

The serine/threonine protein kinases, Unc-51 like autophagy activating kinase (Ulk1/2) are parts of a molecular complex regulating autophagy downstream of mTOR (Walker and Ktistakis, 2019). Rare variants of Ulk1 is associated with schizophrenia (Al Eissa et al., 2018), whilst Ulk2 heterozygous mice show behavioral defects associated with a reduced surface expression of GABA_A_ receptors in pyramidal neurons of the PFC (Sumitomo et al., 2018). In these mice, the autophagy adaptor protein, sequestosome-1 (SQSTM1/p62) was increased, and downregulation of p62 restored the behavior defects and GABARAP mediated localization of the GABA_A_ receptors. Taken together these data show that mTOR plays a role in developing and adult brain to influence autophagy and GABAergic signaling linked to neuropsychiatric disorders (Hui and Tanaka, 2019).

In addition to neurons, alterations in other brain cells such as microglia and astrocytes are also crucial for synaptic functions and plasticity (Vainchtein and Molofsky, 2020). Mice with microglia specific ATG7 deficiency displayed impaired autophagy, defective synaptic pruning, and ASD like behavior defects. Co-cultures of primary neurons with microglia derived from ATG7 deficient mice showed a negative impact on the formation of synapses (Kim et al., 2017). More generally, deficits observed in schizophrenia have been linked to an increased synaptic elimination by microglia (Mallya et al., 2019). Human microglia-like cells derived from schizophrenia patients show an increase in synapse phagocytosis compared to controls (Sellgren et al., 2019). Genetic studies have shown a variant in the complement component 4 (C4) locus associated with schizophrenia (Sekar et al., 2016), and expression of the human C4 gene in mice PFC neurons impaired the development of dendritic spines and affected neuronal connectivity and behavior (Comer et al., 2020). The potential roles of autophagy in the synaptic deficits and neuron-microglia interactions in models of schizophrenia warrant further studies.

## Targeting Autophagy in Neuropsychiatric Disorders

Current strategies to tackle neuropsychiatric disorders by influencing autophagy have mainly centered around mTOR and the use of the inhibitor, rapamycin (Qin et al., 2016; Zhang et al., 2017; Kotajima-Murakami et al., 2019). However, promising data has also been obtained in different models by the use of anti-depressants and antipsychotic drugs (Gulbins et al., 2018; Shu et al., 2019; Lundberg et al., 2020), specific compounds like sphingomyelin synthases inhibitor—tricyclodecan-9-yl-xanthogenate (D609; Gulbins et al., 2018), NAP (Merenlender-Wagner et al., 2014; Sragovich et al., 2017), folinic acid (Frye et al., 2018), Resveratrol (Wang N. et al., 2019), and the drug, propofol in combination with electroconvulsive therapy (Li et al., 2016). Drug candidates modulating autophagy are outlined in Table 2.

**Table 2:** Drugs targeting autophagy.

Drug	Models	Outcome	Reference
Amitriptyline Fluoxetine	Mouse model for depression. Rat pheochromocytoma PC-12 cells.	Increased autophagy regulated by sphingomyelin-ceramide.	Gulbins et al. (2018)
Sphingomyelin synthases inhibitor—D609
Fluoxetine	Postpartum depression model in mice.	Increased autophagy and BDNF in specific regions of hippocampus.	Tan et al. (2018)
Clozapine	Ketamine induced neuronal stem cell culture model.	Activation of autophagy and reduced apoptosis.	Lundberg et al. (2020)
Clozapine	Map6+/− mouse model.	Reduced hyperactivity and no effect on cognition.	Merenlender-Wagner et al. (2014)
NAP	Map6+/− mouse model. SH-SY5Y human neuroblastoma cells.	Combination treatment with Clozapine, reduced hyperactivity and improved cognition. Restoration of Beclin-1. Amelioration of Clozapine induced cell toxicity.
Clozapine Haloperidol	Primary neuronal cultures.	Inhibition of autophagosome fusion with lysosomes.	Park et al. (2012)
Olanzapine	SH-SY5Y human neuroblastoma cells.	Mitochondrial damage, Oxidative stress and organelle autophagy.	Vucicevic et al. (2014)
Clomipramine	Primary neuronal cultures, Mouse models, *C. elegans*.	Inhibition of autophagy.	Cavaliere et al. (2019)
Propofol	Rat model for depression.	Inhibition of autophagy induced by electroconvulsive shock Improvement of learning and memory.	Li et al. (2016)
Rapamycin	Valproic acid rat model of autism.	Improved behavior, induction of BDNF and Bcl2 expression in the hippocampus.	Zhang et al. (2017)
Rapamycin	Valproic acid rat model of autism.	Inhibition of mTOR, increased autophagy and improved behavior.	Qin et al. (2016)

Anti-depressants, amitriptyline, and fluoxetine treatment in mice models of major depressive disorder-induced autophagy secondary to the accumulation of ceramide in the ER (Gulbins et al., 2018). Fluoxetine increased autophagic responses, and the clearance of damaged mitochondria in mice model of depression and astrocyte cultures (Shu et al., 2019). Fluoxetine specifically reduced depressive behavior and increased levels of autophagy proteins and BDNF in a model of postpartum depression in mice (Tan et al., 2018). The antipsychotic drug, clozapine ameliorated autophagy defects, and apoptosis induced by ketamine in neuronal stem cell cultures (Lundberg et al., 2020). Clozapine also promotes neurogenesis *in vivo* but the relative contributions of cell proliferation, cell survival, and autophagy regulation in the action of this drug remain to be established.

Activity-dependent neuroprotector homeobox protein (ADNP) is a microtubule-associated protein linked to neurite outgrowth and autophagy. Post-mortem brain samples from patients with schizophrenia showed a reduction in the ADNP mRNA (Merenlender-Wagner et al., 2015). NAP (NAPVSIPQ) is a peptide derivative of ADNP (Sragovich et al., 2017). Administration of NAP to Microtubule Associated Protein 6 (Map6) gene-deficient mice, increased Beclin-1 expression, and improved the clinical outcome in combination with clozapine (Merenlender-Wagner et al., 2014). Previous findings further suggest that clozapine and haloperidol inhibit the fusion of autophagosomes with lysosomes in primary neuronal cultures (Park et al., 2012). The antidepressant Clomipramine inhibited autophagy in models of primary cortical cultures and *in vivo* mice (Cavaliere et al., 2019). Olanzapine increased mitochondrial autophagy in human neuroblastoma cells as a response to mitochondrial damage and the drug showed increased toxicity upon inhibiting autophagy (Vucicevic et al., 2014).

Taken together these findings show that common anti-psychotics and antidepressants target autophagy in models of neuropsychiatric diseases, but the mechanisms may vary and will require further studies.

## Conclusions and Perspectives

Autophagy and the UPR are two key signaling processes in neuronal physiology whose dysfunctions can underlie the development and manifestation of neuropsychiatric disorders. Recent advancements have shown the importance of protein and organelle quality control and intact synaptic protein turnover in neuronal health. The precise mechanisms, however, by which UPR and autophagy signaling contribute to neurological diseases are still to be explored.

Drugs utilized in clinical therapies, like the anti-depressants and mood stabilizers, have been shown to influence protein handling in neurons in different models. However, the effects of their long-term use need to be studied more carefully, as the drugs might have context and cell-dependent effects that could make their usage a double-edged sword. Along with this, it is still unclear whether the observed responses to the drugs are a part of an adaptive UPR or chronic ER stress resulting in decreased cell viability.

Additional studies with different drugs using cell cultures and animal models of neuropsychiatric disorders are required to assess their mode of action on autophagy and UPR, both in a short time window and more chronic condition. Moreover, the drugs should be studied both on neurons and non-neuronal cells to account for possible cell-specific effects that could be of benefit in treatments. These strategies would further aid in translating the identified mechanisms to viable therapeutic strategies that could be clinically helpful for patients afflicted by neuropsychiatric disorders.

## Author Contributions

All authors contributed to the article and approved the submitted version.

## Conflict of Interest

The authors declare that the research was conducted in the absence of any commercial or financial relationships that could be construed as a potential conflict of interest.

## Abbreviations

4-PBA: 4-phenylbutyrate
ADNP: Activity-dependent neuroprotector homeobox protein
ADHD: Attention deficit hyperactivity disorder
ALS: Amyotrophic Lateral Sclerosis
AMPA: α-amino-3-hydroxy-5-methyl-4-isoxazolepropionic acid
ASD: Autism spectrum disorders
ATF6: activating transcription factor 6
ATG5: Autophagy related 5
ATG8: Autophagy related protein 8
ATG7: Autophagy related protein 7
ATF4: activating transcription factor 4
BDNF: Brain-derived neurotrophic factor
Bip/Grp78: Binding-immunoglobulin protein/78 kDa glucose-regulated protein
cAMP: Cyclic adenosine monophosphate
C4: Complement component 4
CHOP: C/EBP Homologous Protein
CASPR2: Contactin-associated protein-like 2
eIF2α: Eukaryotic initiation factor 2α
eEF2: Eukaryotic elongation factor 2
ER: Endoplasmic reticulum
ERSE: ER stress response elements
ERAD: ER-associated degradation
FXS: Fragile X syndrome
GABA: Gamma-aminobutyric acid
GABARAP: GABA_A_ receptor–associated protein
GADD34: Growth arrest and DNA damage-inducible protein
GRP94: 94 kDa glucose-regulated protein
HD: Huntington’s disease
IRE1α: Inositol-requiring protein 1α
IP3R: Inositol 1,4,5-trisphosphate receptor
LTP: Long-term potentiation
mTOR: Mammalian target of rapamycin
mTORC1: Mammalian target of rapamycin complex 1
Nrf2: Nuclear factor erythroid 2-related factor 2
NGF: Nerve growth factor
PERK: Protein kinase RNA-like endoplasmic reticulum kinase
PD: Parkinson’s disease
PKA: Protein Kinase A
PFC: Prefrontal cortex
PKC: Protein Kinase C
PSD-95: Postsynaptic density protein 95
p75NTR: p75 Neurotrophin receptor
RGS10: G-protein signaling protein 10
SNP: Single nucleotide polymorphism
S1P and S2P: Site-1 and Site-2 proteases
S1R: Sigma-1 receptor
SHANK-3: SH3 and multiple ankyrin repeat domains 3
SQSTM1/p62: Sequestosome-1
TrkB: Tropomyosin receptor kinase B
Tsc2: Tuberous Sclerosis Complex 2
TRIM32: Tripartite motif protein 32
Ulk1/2: Unc-51 like autophagy activating kinase 1/2
UPR: Unfolded protein response
WFS1: Wolfram syndrome protein
XBP1: X-box binding protein 1
XBP1s: spliced X-box binding protein 1
XBP1u: unspliced X-box binding protein 1.
